# Duration of central venous catheter placement and central line-associated bloodstream infections after the adoption of prevention bundles: a two-year retrospective study

**DOI:** 10.1186/s13756-022-01131-w

**Published:** 2022-07-15

**Authors:** Vassiliki Pitiriga, John Bakalis, Elsa Kampos, Petros Kanellopoulos, George Saroglou, Athanasios Tsakris

**Affiliations:** 1 Department of Microbiology, Medical School, National and Kapodistrian University of Athens, 75 Mikras Asias Street, 11527 Athens, Greece; 2 Department of Internal Medicine, Metropolitan Hospital, 9 Ethnarchou Makariou Street, 18547 Athens, Greece

**Keywords:** Catheterization, Central venous catheter, Sepsis, Colonization, Bloodstream infection, Insertion site, Central line-associated bloodstream infection, Peripherally inserted central catheter

## Abstract

**Background:**

Central line–associated bloodstream infections (CLABSIs) remain a critical and possibly fatal outcome of hospitalization. Use of central venous catheter (CVC) bundles can considerably reduce CLABSI rates in hospitalized patients. However, despite widespread adoption of these bundles in hospitals worldwide, CLABSIs still remain prevalent. The aim of the present study was to determine whether longer duration of CVCs placement is related to CLABSIs in hospitalized adults, despite the implementation of preventive bundles. Also to analyse CLABSI pathogens distribution and antimicrobial resistance profiles in different time intervals of catheterization.

**Methods:**

A retrospective study was performed among hospitalized patients who had a CVC inserted during a 24-month period (May 2017–May 2019) and developed CLABSIs. To evaluate the association between CVC placement duration and CLABSI events, we categorized events into three groups, each representing a 10-day time interval.

**Results:**

A total of 59 CLABSI cases were identified among 9774 catheter/days. The CLABSI incidence rate per 1000 catheter/days was 4.80 for duration of catheterization up to 10 days, 5.92 for duration of 11–20 days, and 8.64 for duration > 20 days(*p* = 0.007). The CLABSI incidence rate per 1000 catheter/days due to multidrug-resistant organisms (MDROs) among the three groups was 2.62 for catheter duration of up to 10 days, 3.83 for 11–20 days, and 3.46 for > 20 days (*p* = 0.14). Among CLABSIs, the most common microorganism identified was multidrug-resistant *Acinetobacter baumannii*, which accounted for 27.1% of the cases. There was no significant difference in the type of CLABSI pathogens isolated among the 3 groups.

**Conclusions:**

Our findings suggest that duration of CVC placement remains an important risk factor for CLABSIs in hospitalized patients, even after the adoption of prevention bundles. The high prevalence of MDROs in our setting reflects the local epidemiology, highlighting a significant threat of urgent public health concern.

## Introduction

Central line-associated bloodstream infections (CLABSIs) remain important healthcare-associated infections leading to prolonged hospital stays and increased healthcare costs and mortality [1, 2]. Identified CLABSI risk factors before the adoption of preventive bundles include prolonged hospitalization before catheterization, femoral catheterization, longer catheterization duration, use of total parenteral nutrition, extensive catheter manipulations, and reduced nurse: patient ratio [3]. The implementation of central venous catheters (CVCs) bundles including avoidance of femoral sites for CVC insertion, strict adherence to hand hygiene protocols, use of full barrier precautions, chlorhexidine skin preparation, and removal of unnecessary catheters, considerably decreased CLABSI rates in hospitalized patients [4]. However, CLABSIs rates still remain at least 1 case per 1000 catheter-days in US hospitals, despite the broad and continuous implementation of these bundles [5].

With regards to the duration of catheterization, current guidelines recommend that catheter removal is required only if it is no longer needed [6]. On the basis of this recommendation, many patients who have limited vascular access and need prolonged parenteral treatment use CVCs for periods longer than 3 weeks, raising the concern for developing CLABSI events despite the implementation of CVC hygiene bundles. The existing literature on CLABSI prevention measures has yielded varying and opposing suggestions on the appropriate duration of catheterization, with some recommending optimal cut-offs to avoid CLABSIs and others supporting that an indefinite duration may be acceptable [7–9].

Most of the previous studies that examined the catheter duration as a risk factor for CLABSIs were performed in paediatric populations that mainly concerned peripheral inserted central catheters (PICCs) since they are more commonly used by clinicians in daily practice [8, 9].

The aim of the present study was to examine the association between duration of CVC placement and CLABSI rates in hospitalized adults during a period of prevention bundles adoption. The study was conducted in the context of investigating increased rates of CLABSIs in a tertiary large hospital with highly trained personnel. For this purpose, we categorized events into consecutive 10-day time intervals, divided into 3 groups. We also analysed and compared distribution of CLABSI pathogens and their resistance profiles between the three groups.

## Materials and methods

We performed a retrospective analysis of data collected from consecutive admissions to Metropolitan Hospital, a large tertiary care hospital of Piraeus, Attica Prefecture, covering a 24-month period from May 2017 to May 2019. This observational study was approved by the institutional review board.

### Data collection

After insertion, catheters were checked using a check-box form containing the patient’s diagnosis, operator’s name, site chosen, date placed and removed, date of intensive care units (ICU) discharge or death, mechanical ventilation, arterial catheters, parenteral nutrition, and daily clinical assessment (e.g., discharge, erythema, and tenderness) of possible catheter infection. The operator inserting the catheter entered the initial data; nurse personnel entered data the following days while the infection control nurse monitored data collection 3–4 times per week. We retrospectively collected study data from three different sources where information was completed: 1) ICU database (for demographic and clinical data related to the patient’s admission and clinical course); 2) and 3) Clinical Laboratory and hospital infection control team database (for blood culture and antibiotic susceptibility results).

### Catheterization protocol

In our hospital triple lumen, non-antibiotic impregnated catheters (Arrow model, total provided by Arrow^®^/Teleflex^®^, Wayne, USA) are mainly used. Double lumen catheters (Arrow^®^/Teleflex^®^, Wayne, USA), are also used but in a lower percentage, particularly in patients that do not require complex therapeutic interventions. The choice of the site of insertion was left to the discretion of the physician caring for the patient. Maximal sterile barrier precautions (large sterile drape; surgical hand antisepsis; and mask, cap, sterile gloves, and gown) were used at catheter insertion according to CDC recommendations.

### Catheter care protocol

Standardized CVC care practices were implemented by a highly proficient nursing staff. Every couple of days or earlier if clinically required, the nursing staff changed the dressing, cleaned the skin site and the catheter hub with iodine solution, and changed the intravenous accessory tubing. CVCs were removed when (a) there was evidence or suspicion of infection, (b) when the catheter was no longer required.

### Culture techniques

All catheters were examined for the presence of pathogens either as a routine after removal or after suspicion of infection. After disinfecting skin around the CVC entry site, the proximal 4–5 cm part of the tip was cut off using sterile scissors. The specimen was placed in a sterile container and transported to the microbiology laboratory within 15 min at room temperature. The intradermal and intravascular portion of the catheter was analyzed by the semiquantitative culture technique described by Maki et al. [10]. According to Maki’s technique, catheter-tip culture is considered positive in the presence of ≥ 15 colony-forming units (CFU) growth of any organism.

Blood cultures were incubated in Becton Dickinson Bactec (BD Bio-sciences, USA) in aerobic and anaerobic broth media. Identification of isolates and antimicrobial resistance patterns were determined by the VITEK^®^2Automated Compact System (BioMérieux Co., France). E-test (BioMérieux Co., France) was performed as an additional test, in order to confirm the resistance phenotypes reported by the VITEK System, according to the standard laboratory procedures.

### Definitions

Catheter infection and colonization definitions were based on the Centers for Disease Control bloodstream infection guidelines and the semi-quantitative culture technique by Maki et al.

*Catheter associated BSI (CLABSI)* was defined as a laboratory confirmed BSI (a positive blood culture with no other apparent source of infection) occurring in the presence of a CVC or within 48 h of CVC removal.

*Catheter/days* was defined as the number of CVCs presents among all units’ patients at 08:00 h each morning. When more than one concurrent CVC was present in the patient, they were counted as one CVC.

*Multidrug-resistant organisms (MDROs)* were defined as **s**pecies of microorganisms that exhibit antimicrobial resistance to at least one antimicrobial drug in three or more antimicrobial categories. This definition concerns both gram-positive and gram-negative bacteria [11].

### Statistical analysis

Descriptive analysis to characterize patients’ population were reported as count (percent) or mean value (+ / − standard deviation) for qualitative and quantitative variables, respectively, and were compared between the three groups using Chi-square or one-way ANOVA test, as appropriate. A *p*-value of ≤ 0.05 was considered as statistically significant.

## Results

A total of 9774 catheter/days were reported and analyzed during the 2-years period. Among them, a total of 59 CLABSI cases were identified. The total patients’ demographic characteristics are presented in Table 1. Distribution of CLABSI events among hospital units was as follows: Oncology Unit 3%; ICU 43%; Surgery Unit 14%; Pathology Unit 35%; other units 5%. The mean duration of catheter placement was 16, 2 ± 10.1 days (Range: 2–56 days). In order to evaluate the association between CVC duration and incidence rate of CLABSIs, we categorized events into three groups, each representing a 10-day time interval of catheterization. In the group of up to 10 days (group 1) 22 CLABSI cases were included, 17 cases in the group of 11–20 days’duration (group 2) and 20 cases in the group of > 20 days’ duration (group 3). The mean age of the participants in each group was 52.7 ± 16.7 for group 1, 63.3 ± 22.7 for group 2 and 50.6 ± 19.2 for group 3 (ANOVA, *p* = 0.1). The male/female analogy was 14/8 in the group 1, 12/5 in group 2 and 15/5 in group 3 (X^2^, *p* = 0.72). No significant differences in demographic characteristics were determined among the 3 groups (data not shown). No differences also existed in the proportion of catheterization site (femoral, internal jugular and subclavian) among the 3 groups (Table 2).

**Table 1:** Study populations’ demographic and clinical characteristics

Variable	CLABSI patients (*n* = 59)
	*N* (%)
Age, mean ± SD, (years)	55.08 ± 19.8
Gender (M/F)	41/18
Obesity	19 (32.2)
Diabetes mellitus	9 (15.2)
Pulmonary disease	16 (27.1)
Hypertension	11 (18.6)
Renal disease	17 (28.8)
Oncologic disease	16 (27.1)
Immune deficiency/suppression	17 (28.8)
Admission category	
Medical	43 (72.8)
Surgery	16 (27.1)
Mechanical ventilation	37 (62.7)
Cardiovascular disease	17 (28.8)
Neurological disease	38 (64.4)
Gastroenterological disease	18 (30.5)
Hospital death	19 (32.2)
Sepsis	11 (18.6)
APACHE score	12.8 ± 8.2
Length of catheter stay (mean ± SD)	16.19 ± 10.7

*N*, *n* number, *SD* standard deviation

**Table 2:** Site of catheter insertion among the CLABSI groups

	Mean (days) ± SD	2–10 days	11–20 days	> 20 days	*P–*value
Femoral (*n* = 13)	15.2 ± 16.1	4/19%	5/25%	4/22.2%	X^2=^ 1.57 *P* = 0.81
Internal jugular (*n* = 30)	15.7 ± 8.3	12/57.1%	8/40%	10/55.6%	
Subclavian (*n* = 16)	17.8 ± 10.1	5/23.9%	7/35%	4/22.2%	

*p* ≤ 0.05 significant

The CLABSI rates were 4.80 in group 1, 5.92 in group 2, and 8.64 in group 3 (ANOVA, *p* = 0.007). The CLABSI rate due to multidrug-resistant organisms (MDROs) among the 3 groups was 2.62 in group 1, 3.83 in group 2, and 3.46 in group 3 (ANOVA, *p* = 0.14) (Table 3).

**Table 3:** Incidence rate of CLABSIs and MDROs among the CLABSI groups

	2–10 days	11–20 days	> 20 days	*p–*value
No of catheters	904	202	81	
Cath/days	4.585	2.874	2.315	
CLABSI (*n* = 59)	22	17	20	X^2^ = 84 *p* = 0.001
CLABSI, %	2.43%	8.42%	24.69%	X^2^ = 25.9 *p* = 0.001
CLABSI, incidence rate (per 1,000 cath/days)	4.80	5.92	8.64	ANOVA, F = 7.61 *p* = 0.007
MDROs (*n* = 31)	12	11	8	X^2^ = 29.4 *p* = 0.001
MDRO, %	1.3%	5.4%	9.9%	X^2^ = 8.05 *p* = 0.01
MDRO incidence rate (per 1.000 cath/days)	2.62	3.83	3.46	ANOVA, F = 1.05 *p* = 0.144

*p* ≤ 0.05 significant

Within CLABSI events, the most common microorganism identified was MDR *Acinetobacter baumannii* (*n* = 27.1%). Among the 3 groups, MDR *A. baumannii* was the predominant pathogen in group 1 (36.4%), MDR *K. pneumoniae* (35.3%) for group 2 and both pathogens equally for group 3 (20%) (Table 4). No significant difference in the type of isolated CLABSIs pathogens between the 3 groups was detected.

**Table 4:** Pathogen distribution among the CLABSI groups

Gram–negative bacteria	CLABSIs, *n* (%)			
	< 10 days	11–20 days	> 20 days	Total
MDR *K. pneumoniae*	3 (13.6)	6 (35.3)	4 (20)	13 (22)
MDR *A. baumannii*	8 (36.4)	4 (23.5)	4 (20)	16 (27.1)
MDR *P. aeruginosa*	1 (4.5)	1 (5.9)	–	2 (3.4)
Non–MDR *P. mirabilis*	–	–	1 (5)	1 (1.7)
Non–MDR *P. aeruginosa*	1(4.5)	–	–	1 (1.7)
*Gram positive bacteria*				
*Staph. coagulase neg*	4 (18.2)	2 (11.8)	3 (15)	9 (15.3)
Methicillin–resistant *S. aureus*	1 (4.5)	–	2 (10)	3 (5.1)
*Enterococcus* spp*.*	–	–	2 (10)	2 (3.4)
*Yeasts*				
*Candida* spp.	3 (13.6)	–	3 (15)	6 (10.2)
*Other bacteria*				
*Pseudomonas stuartii*	1 (4.5)	–	–	1 (1,7)
*Stenotrophonas maltophilia*	–	2 (11.8)	–	2 (3.4)
*Serratia marcescens*	–	1 (5.9)	1 (5)	2 (3.4)
*Streptococcus* spp*.*	–	1 (5.9)	–	1 (1.7)

## Discussion

CVCs are important in managing many clinical practices such as blood sampling and infusion of medications, particularly in ICUs. Therefore, in the real world setting, a CVC can remain even for months [12, 13]. Prior to the widespread implementation of CVC bundles, longer duration of catheterization was listed as an identified and significant CLABSI risk factors among others such as use of total parenteral nutrition and extensive catheter manipulation [14]. However, these risk factors refer to practices from a long-gone period [15].

In our setting, CLABSI prevention bundles were adopted in 2009, and included avoidance of femoral sites for CVC insertion, strict adherence to hand hygiene, use of full barrier precautions, chlorhexidine skin preparation, and removal of unnecessary catheters. However, despite the widespread adoption of these bundles and high compliance, CLABSIs still continued to occur in rates that were not negligible. Therefore, we conducted the present study to further investigate the risk factors for these events by analyzing the clinical characteristics of patients, focusing on duration of catheters maintenance and the pathogen distribution associated with CLABSIs after the bundle adoption. Certain limitations should be acknowledged at this point. They mainly concern the short study time-period and the small sample size. More specifically, we present an observational study through a retrospective analysis of two years’ period-due to the lack of complete epidemiological data from previous years- in which only 59 CLABSI cases were identified and analyzed. However, despite these limitations, we have managed to yield additional valuable information that will provide the opportunity for future modifications of our hospital’s CLABSI prevention bundles.

Our analysis showed that an increased duration of central venous access was associated with a gradually higher rate of CLABSI events, in a statistically significant level. In fact, although an increase in CLABSI rate was already noticed during the first ten days of catheterization (group 1), the rate value had almost doubled after 20 days (group 3). Overall, there was no significant difference in patients’ clinical characteristics, including sex, age, underlying diseases, risk factors for CLABSI, laboratory findings, or clinical outcomes, between the 3 groups.

We have considered time as a categorical variable to assess the existing risk over the duration of the catheterization, similarly to other studies [16]. Taking into consideration the existing bibliography referring to the selection of appropriate time thresholds for catheter replacement [17, 18], we set cut-points at 10 days following catheter insertion for each of the 3 groups [19].

Our study indicated that A*cinetobacter* spp*., K. pneumoniae,* coagulase-negative staphylococci and *Candida albicans* were the most common microorganisms isolated from CVCs. *Acinetobacter* is an important nosocomial pathogen that can be found in many health care environments, on the environment and hands of ICU staff. It frequently colonizes hospitalized patients, especially those with mechanical ventilation in ICU and patients with indwelling catheters [20]. Similarly, to our findings, another retrospective study in an adult ICU in a tertiary care hospital has also showed that the most frequently isolated organism was *A. baumannii* [21].

In consistence with the international CLABSI pathogen distribution patterns available from numerous studies, causative microorganisms typically originate from the normal resident flora of the skin present at the insertion site, which are mostly consisting by gram-positives such as S*taphylococcus* spp*, Streptococcus* spp*., Corynebacterium* spp*.,* and *Candida* spp*.*[22–24].However, later studies and data from our previous survey, support that CLABSIs caused by Gram-negatives either predominated in the panel of isolated organisms or exhibited growing trends [25–27]. In our study the epidemiology profile of CLABSI pathogens reflects the recently published Greek ICU pathogen profile, where MDR *A. baumannii* is frequently isolated [28]. This emergence of MDR pathogens has created a great concern on medical care in Greek hospitals, especially for ICU patients [29].

Based on the annual data of antimicrobial resistance rates reported by our hospital’s clinical laboratory, the rates of the 3 most commonly isolated MDR Gram-negatives (*A. baumannii, K. pneumoniae, P. aeruginosa*) isolated from nosocomial patients was 21.6% for the same time period, 63.3% of which were isolated from ICU. Moreover, 43% of CLABSIs by MDROs were recovered from ICU patients, whereas all patients from other hospital units had a history of previous admission to our ICU prior to the MDROs isolation. This fact, combined with the endemicity of Gram-negative MDROs in our hospital has led to the domination of these pathogens in the microbial profile of CLABSIs.

## Conclusions

The findings of the present study displayed that longer duration of catheter placement was associated with an increase in CLABSI rates, supporting the need for continuous training of personnel regarding the proper implementation of bundles, continuous measurement of compliance indicators and probably reassessment of the bundles strategy. Most attention should be focused not only on improving catheter insertion procedures but also and most importantly on maintenance of anaseptic setting during the postinsertion care of the CVCs, a period that seems most likely that infection prevention lapses occur.

In patients with a CVC who develop a CLABSI, the catheter is regularly removed, especially if the causative pathogen is a Gram-negative pathogen or *Candida*. On this basis, an approach of preventive catheter replacement if intravascular access beyond a specific time period is required, should be considered necessary. However, future studies should determine the total cost–benefit of preventive catheter replacement and the optimal time cut-offs. Moreover, a substantial swift in the epidemiological profile of CLABSIs pathogens towards a high proportion of Gram-negative pathogens and specifically MDROs was noted. Since multidrug-resistant Gram-negative infections are associated with considerable mortality, empirical treatment should be focused on their increasing prevalence and be directed by regional epidemiology reports.

## Data Availability

All data generated or analyzed during this study are included in this published article.

## Abbreviations

CLABSI: Central-line associated bloodstream infection
CRI: Catheter-related infection
CVC: Central venous catheter
ICU: Intensive care unit
MDRO: Multidrug-resistant organism
